# A review of the physiology behind letrozole applications in infertility: are current protocols optimal?

**DOI:** 10.1007/s10815-020-01892-6

**Published:** 2020-07-26

**Authors:** Bruce I. Rose, Samuel E. Brown

**Affiliations:** 1 Brown Fertility, 8149 Point Meadows Way, Jacksonville, FL 32256 USA; 2 Department of Obstetrics and Gynecology, School of Medicine, University of Central Florida, Orlando, FL 32816 USA; 3 Department of Obstetrics and Gynecology, School of Medicine, University of Florida, Jacksonville, FL 32209 USA; 4 School of Medicine and Science, Department of Obstetrics and Gynecology, Mayo Clinic, FL 32224 Jacksonville, USA

**Keywords:** Letrozole, Aromatase inhibitor, Androgens, Ovarian physiology, Ovulation induction, Fertility preservation, Diminished ovarian reserve, In vitro maturation, IVM

## Abstract

Letrozole is a targeted aromatase inhibitor which has primarily been used in post-menopausal women with breast cancer. Recently, it has been utilized in infertile pre-menopausal women because of its ability to enhance FSH production for ovulation induction. However, the ovarian follicle’s response to FSH is only a part of the endocrine events occurring in a developing follicle. The health of the small antral follicles is driven primarily by androgens, which contribute to granulosa cell mitosis, sensitivity to FSH, and resistance to atresia. In contrast, elevated androgens in the late antral to pre-ovulatory follicle have a negative impact on follicle health and lead to atresia and cystic follicle formation. This ovarian physiologic data suggests that current applications of letrozole to infertility may be squandering some of the primary benefits available in using letrozole to promote follicle development. Four applications of letrozole to infertility that have appeared in the medical literature are reviewed. Androgen-related benefits are reviewed and various questions put forward about how letrozole could be more effectively used to help patients in these settings.

## Introduction and background

Letrozole has been used with increasing frequency in infertility therapy. Letrozole blocks the conversion of C-19 androgens to C-18 estrogens by competitively inhibiting the enzyme, aromatase (cytochrome P-450 19), which is an essential step in estrogen biosynthesis in the ovary and other tissues. The use of letrozole results in increased androgens and decreased estrogens in tissues throughout the body [1, 2]. Letrozole has proven to be a valuable drug in the treatment of estrogen receptor–positive breast cancers which constitute about two-thirds of all breast cancers [3, 4].

In this mini-review, we examine four applications of letrozole to treat infertility that have appeared in the medical literature. The focus of this review is on how these therapies utilize the pharmacological properties and physiological ovarian consequences of letrozole. The initial use of letrozole in infertility mimics the long-term use of clomiphene citrate in infertility therapy. Clomiphene citrate was used to increase FSH secretion from the pituitary by blocking a negative estradiol feedback loop by binding to estradiol receptors in the hypothalamus. Contemporary applications of letrozole to infertility use the same negative feedback loop to increase FSH secretion, but do so by decreasing estradiol production from the ovary and other tissues.

Infertility applications of letrozole are quite different from the primary medical application of letrozole to treat breast cancer. Aromatase inhibitors are used in post-menopausal women with breast cancer, who have a (relatively) long duration of planned treatment. The impact of letrozole use in a setting of rapid endocrine shifts requires a more subtle assessment of its impact. Letrozole effects follicle development, follicle growth, and follicle atresia. These effects on a given ovarian follicle are complex, because they vary depending on where that follicle is in its growth trajectory and on what other drugs are being used at the same time. Theoretically, using letrozole solely to enhance FSH secretion is to only utilize a portion of its impact on the ovary. Little information is presently available on whether or not it is clinically useful to try to incorporate non-FSH impacts of letrozole on the ovary into infertility therapies.

The goal of this paper is to first review clinically important pharmacological aspects of letrozole. We will then review follicular physiology and endocrine activity as a follicle changes from a pre-antral to a pre-ovulatory follicle. We will further review some of the impacts of the endocrinological changes induced by androgens on granulosa cell apoptosis and antral follicle atresia. Finally, we will look at four selected applications of letrozole (see Table 1) from the infertility literature to see how these applications use letrozole or how they could more fully utilize the impact of letrozole on ovarian physiology.

**Table 1:** The four selected applications of letrozole

Letrozole application	Letrozole dose and timing	Selected literature references
Oral ovulation induction for fertility enhancement in the ovulatory patient	Letrozole 2.5 to 7.5 mg on days 3 through 7 of menstrual cycle	[5–7]
IVF in patients with breast cancer needing fertility preservation	Letrozole 5 mg per day starting on day 2 or 3 of menstrual cycle followed by gonadotropins 2 days later until triggered for maturation	[8, 9]
IVF in patients with diminished ovarian reserve	2.5 to 5 mg for 5 days starting simultaneously with high dose FSH during antagonist IVF cycle	[10, 11]
Priming for in vitro maturation (IVM) patients	2.5 to 5 mg on days 3–7 of menstrual cycle with low dose FSH started on day 5	[12, 13]

### Letrozole pharmacology

First-generation aromatase inhibitors have been available since the 1970s, but, although they blocked estrogen biosynthesis, they also inhibited the biosynthesis of cortisol, aldosterone, and thyroid hormone [14]. An additional problem was liver toxicity. Second-generation aromatase inhibitors were safer and more potent, but were still not adequately selective in inhibiting estrogen production. Third-generation aromatase inhibitors became available in the 1990s. They were created utilizing new molecular modeling techniques designed to enable better binding to the active site in aromatase. Letrozole was one of the molecules created that met the goal of high potency and selectivity. Letrozole and anastrozole are clinically available non-steroidal (i.e., type II) third-generation aromatase inhibitors.

Letrozole has 99.9% bioavailability after oral administration. It has a single dose terminal half-life of 42 h, but a steady-state concentration is not achieved for 2 to 6 weeks (steady-state half-life is 118 h) [1, 15]. This is thought to be because letrozole is metabolized into inactive metabolites by two cytochrome P-450 enzymes: 3A4 and 2A6. Cytochrome P-450 2A6 has a much higher affinity (and is a more efficient metabolizer) to letrozole than 3A4, but becomes saturated at a letrozole dose of about 2.5 mg [1, 15].

Estrogen suppression by letrozole has been looked at in many in vitro settings, for example, human placental tissue, hamster ovarian tissue, various cancer cell lines, human breast tissue, human adipose fibroblasts, rodent cells, and particulate breast cancer tissue [1]. In these settings, letrozole is highly efficient in suppressing estrogen production. Consequently, letrozole is an effective drug for the treatment of post-menopausal women with breast cancer [4, 15]. Letrozole was not designed for short-term use in pre-menopausal women and there is relatively limited direct research on how it impacts the ovarian follicle throughout its development.

Aromatase is a member of the cytochrome P450 superfamily which contains over 480 members. Aromatase is found in tissues throughout the body and the regulation of its expression is by tissue-specific promoters and activators. In the ovary, cAMP and gonadotropins regulate aromatase expression [16]. Significant aromatase expression does not occur in granulosa cells in follicles until they are about 9 mm in diameter and starts to markedly accelerate in 10-mm follicles [17–19]. However, where ever aromatase occurs, it is highly specific for binding androgens, and, by design, letrozole. If testosterone docks with an aromatase molecule in ovarian tissue, it is rapidly converted to estradiol, which has only a weak affinity for it and the estradiol molecule is quickly released into the cytoplasm. The aromatase binding site is then free to competitively dock with either an androgen or a letrozole molecule. Letrozole circulates with 55% bound to albumen and the impact of its pharmacology is that aromatase activity is decreased by more than 99% (at a dose of 2.5 mg) [1, 15, 16]. In the pre-menopausal woman treated for infertility with letrozole, estradiol can still be produced during the follicular phase of the menstrual cycle, either because of a reduction in letrozole availability due to letrozole’s half-life, the treatment regimen, or because of enhanced aromatase expression primarily due to increased FSH and FSH receptor availability.

Androgen production is not directly increased by letrozole. In the pre-menopausal woman, intra-ovarian androgen levels are increased, primarily because less androgen substrate is used for estrogen biosynthesis. Ovarian androgen production occurs in the theca (and interstitial) cells in response to LH and, in the large follicle, is augmented by IGF-1. However, in the early follicle, oocyte-derived growth differentiation factor 9 (GFD9) is essential for theca cell differentiation and androgen production [20, 21]. Follicles with poor theca cell development do not develop further.

### Follicular development

**Fig. 1:**
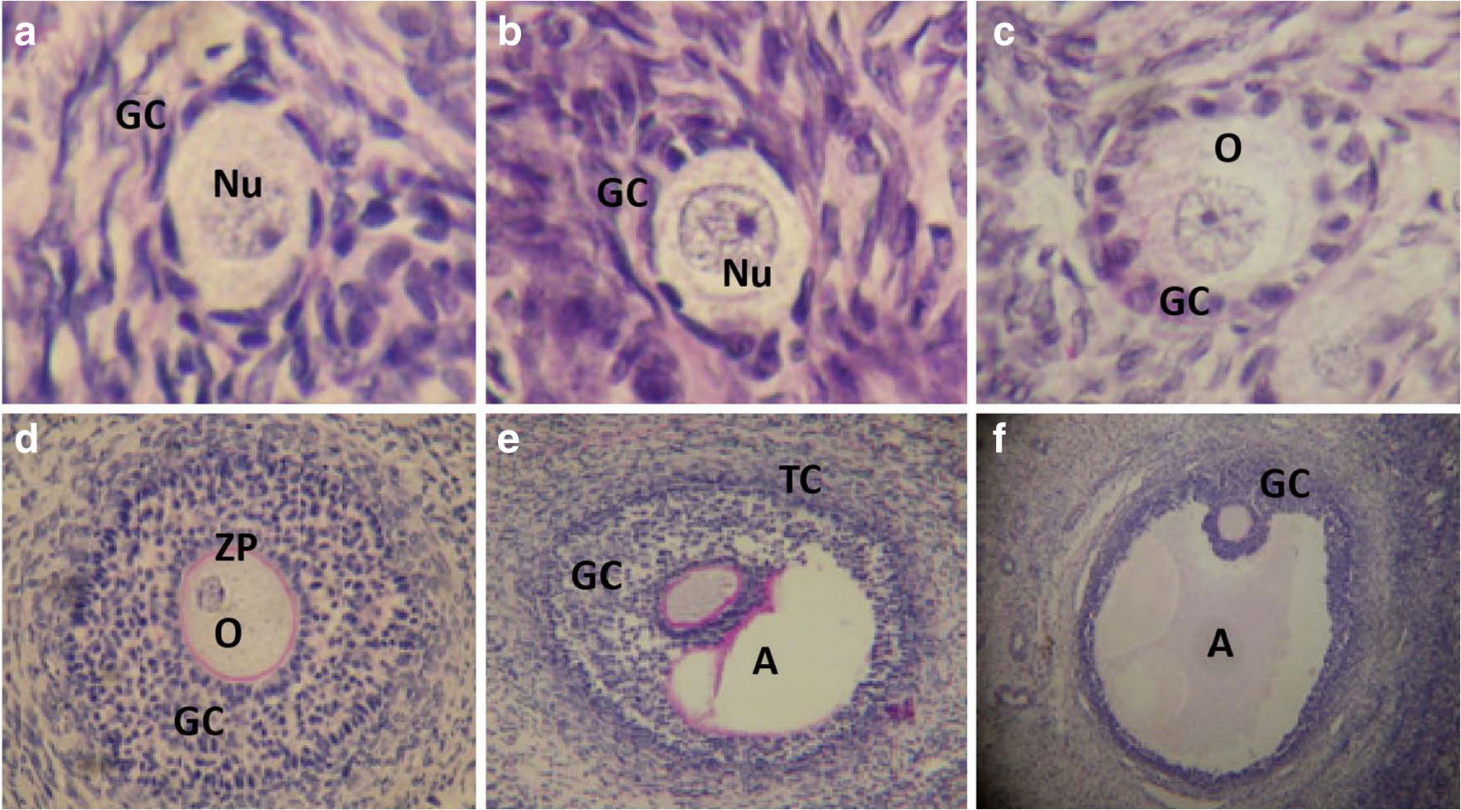
Histological sections showing different stages of follicle development. **a** Primordial follicle; **b**, **c** Primary follicles; **d** Pre-antral follicle; **e** Antral follicle; **f** Pre-ovulatory follicle. Nu, oocyte nucleus; O, oocyte; GC, granulosa cells; ZP, zona pellucida; A, antrum; TC, theca cells. From Celestino et al. Mechanisms of atresia in ovarian follicles. *Anim Reprod* 2009; 6(4): 495–508

**Fig. 2:**
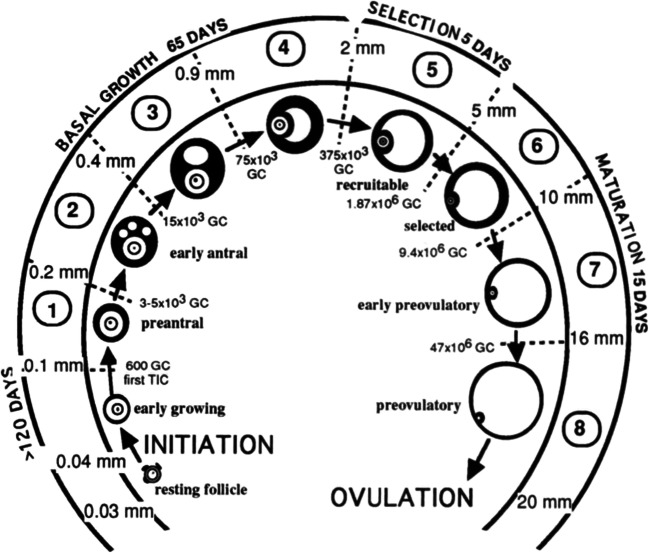
Gougeon’s classification of follicles in the human ovary. From Gougeon. Regulation of ovarian follicular development in primates: facts and hypotheses *Endocrine Rev*. 1996; 17(2): 121–155

**Fig. 3:**
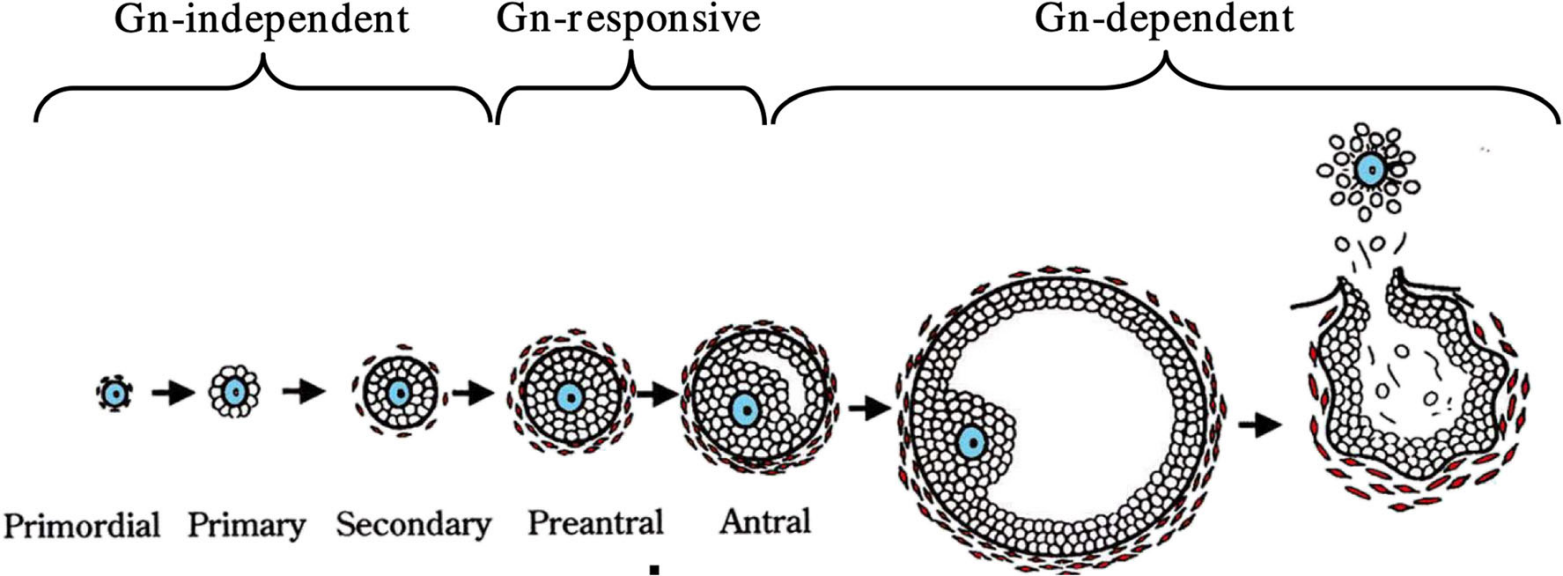
Follicle development emphasizing FSH independence, responsiveness, or dependence. Adapted from Orisaka et al. Oocyte-granulosa-theca cell interactions during preantral follicular development. *J Ovarian Res* 2009; 2: 9

Follicular development in women is a complex event that is challenging to understand at the molecular level. To understand how letrozole affects ovarian physiology, current nomenclature does not fully capture a follicle’s changing molecular endocrine environment. The most widely used nomenclature describing follicle growth divides follicles into primordial, primary, secondary, pre-antral, antral (or tertiary), and pre-ovulatory stages based on unequivocally defined morphological characteristics (Fig. 1). This characterization is not optimal in applying ovarian physiology to infertility applications since most cycle manipulations used occur in the late pre-antral and the antral follicle stages. Gougeon’s characterization of follicle growth in humans defines eight stages of development based on the number of granulosa cells in follicles which also correlates to a follicle’s diameter (Fig. 2) [22]. Gougeon’s characterization is more helpful in understanding infertility interventions in humans, since human follicle diameters are easy to obtain, but they do not directly facilitate specific application of research done in other species (which have different follicle diameters and granulosa cell numbers) to humans; information transfer would require a more physiological characterization of follicle growth. An example of this type of observation, limited to FSH’s significance for follicle growth, is given by Orisaka et al. [23] (Fig. 3). FSH receptors are detectable on granulosa cells in the early pre-antral follicle [17, 23] and thus can respond to FSH several months prior to ovulation. However, the time at which the follicle transitions from being FSH responsive to FSH dependent is clinically important and occurs in all mammals. In the human, this occurs when follicles are between 4 and 6 mm in diameter (Gougeon class 5 to 6). As will be discussed, the transition from peak androgen dependence also occurs around this same time [18, 24].

### Beneficial effects of androgens on small follicles

**Fig. 4:**
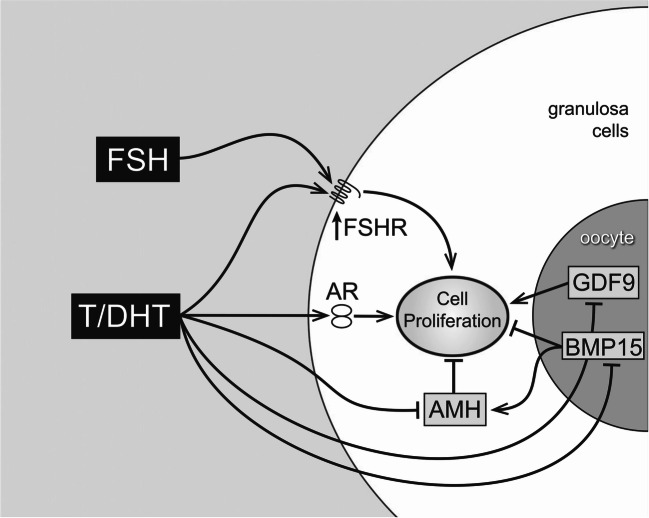
Pathways of androgen action on pre-antral follicle growth. Testosterone or dihydrotestosterone (DHT) acts via the androgen receptors to increase granulosa cell (GC) proliferation. This may be mediated directly or indirectly by increased FSH receptors (stimulating GC mitosis) or decreased AMH (reducing AMH inhibition). AMH is further reduced by androgen-induced reduction of BMP15, which normally stimulates AMH levels. Adapted from Laird et al. Androgen stimulates the growth of mouse preantral follicles in vitro: interaction with follicle-stimulating hormone and with growth factors of the TGFbeta superfamily. *Endocrinol* 2007; 158: 920–935

Studies consistently show that androgens play important positive roles in pre-antral to early antral follicle development [12, 25–29]. In this setting, they promote granulosa cell mitosis [24, 25, 30], increase FSH receptor expression and FSH receptor protein [21, 26, 30, 31], and prevent granulosa cell apoptosis [31]. Although androgens are critical hormones in the pre-antral to early antral follicle’s stage of development, they are not acting alone in promoting follicle growth and development. For example, acting in conjunction with FSH and modulated by IGF-1 and GDF9 (positively) and AMH and BMP15 (negatively), androgens promote granulosa cell proliferation [24] (Fig. 4).

Peak numbers of androgen receptors are found in granulosa cells of human antral follicles between 3 and 6 mm in diameter [18, 19, 24]. Androgen receptors become fewer in granulosa cells of larger antral follicles and are significantly reduced in granulosa cells of early pre-ovulatory follicles [18, 19, 24]. The antrums of human small antral follicles less than 7 mm in diameter are primarily androgenic [18, 32]. During this period of increasing responsiveness to androgen actions through their receptor, the human antral follicle has increased its granulosa cell mass from a few thousand cells to about ten million cells [22, 33]. The follicles, which continue to grow and differentiate to become pre-ovulatory, will need to continue mitosis at this pace to create a granulosa cell mass of about 50 million cells approximately 1 week later. Classical steroid androgen hormone signaling with receptor activation in the cytoplasm, transfer of the activated receptor into the nucleus, and binding of the activated receptor to a gene promoter is less frequent after the follicle grows out of this small antral follicle stage and is almost non-existent in the last stages of follicle growth. The mechanism of granulosa cell mitosis and follicle growth must change from a primarily androgen-driven system.

FSH and estrogen’s actions on granulosa cells become important for extending follicle growth past the early antral follicle transition point [27, 34, 35]. Nevertheless, FSH receptor expression decreases markedly per granulosa cell in human follicles 5–6 mm in diameter and continues to decrease as the follicle diameter increases [18, 19]. In fact, Jeppesen found a 250-fold decrease in FSH receptor expression per granulosa cell from small antral to pre-ovulatory follicles before ovulation [19]. For a dominant (or rescued) follicle to be able to maintain a healthy FSH receptor population to respond adequately to FSH in producing estrogen, it must increase the number of its granulosa cells. FSH induces aromatase expression in granulosa cells and that expression starts to markedly increase in the human 11–13-mm follicle, with a concomitant increase in estradiol, and continues to increase as follicle diameter expands to pre-ovulatory follicle size [19]. More granulosa cells in a follicle lead to more FSH receptors per follicle with increased estrogen production resulting in more mitosis [27, 33]. Estrogen is also essential for LH receptor expression in the granulosa cell, which only starts to be expressed on granulosa cells in an antral follicle greater than 10 mm in diameter and is required for eventual ovulation [24].

Most human atretic follicles have diameters in the 1 to 10 mm in range (Gougeon class 5, 6, and 7) with most atresia occurring in the smaller antral follicles [35]. Gougeon found that the doubling time for granulosa cell mitosis decreased by 50% (from 10 to 5 days) in a 5-mm-diameter human follicle compared with granulosa cell division in slightly smaller follicles [22]. McNatty et al. found that healthy antral follicles contained more than twice as many granulosa cells as atretic follicles of the same size and that granulosa cell number was correlated with the estradiol level in the antral fluid [33]. This suggests that follicles 6–12 mm are at one of the sensitive stages in their destiny, possibly because of this increased requirement for accelerated mitosis. As receptor-mediated androgen benefits taper off, a follicle must be able to use FSH receptor–mediated benefits to continue to grow. This requires healthy follicles to accelerate granulosa cell mitosis, which, as the follicle enlarges, is primarily a consequence of FSH [16, 35, 36].

FSH activity is essential to enable follicles to develop so that they contain a fertilizable oocyte. Estrogen production is essential for granulosa cells to develop LH receptors eventually enabling spontaneous ovulation [37]. FSH works though its G-coupled cell surface receptor on granulosa cells to activate protein kinase A (via cAMP) and subsequently a pathway that leads to phosphorylation and activation of protein kinase B (Akt) [34, 38]. The Akt pathway is a necessary (but not sufficient) activator of many FSH target genes including the LH receptor and aromatase. The Akt pathway works by phosphorylation of many proteins (at least fifty have been identified) resulting in either stimulation or inhibition of various processes [39]. Some of these pathways also require activation of other pathways to achieve a particular action. In particular, Akt phosphorylates members of the forkhead box O (FOXO) family to stimulate mitosis and inhibit apoptosis (by inhibiting expression of a proapoptotic protein) [39–41].

Although androgens are essential for pre-antral and early antral follicle development, the early follicular unit works to restrain androgen production from the theca cells with factors from both the granulosa cells and the oocyte [27]. Androgen excess secondary to letrozole treatment will be limited by the amount of estrogen that the follicular unit is trying to produce (related to the amount of androgen that theca cells are producing). Clinically, it is an open question as to how effective letrozole can be in elevating intra-follicular androgens in these early follicles and if that elevation can be utilized beneficially by the follicle.

### Granulosa cell apoptosis and antral follicle atresia

Apoptosis is a cellular event, whereas atresia is a follicular event. Cellular apoptosis is characterized by loss of cell volume (cytoplasmic condensation), nuclear pyknosis (resulting from margination of the chromatin and redistribution against the nuclear membrane), and cytoplasmic blebbing (formation of membrane-bound vesicles containing intact cytoplasmic organelles). These vesicles or apoptotic bodies are phagocytized by neighboring cells so that cell death and the cell’s elimination from the follicle occur without causing an inflammatory response. A hallmark of apoptosis is an endonuclease-mediated cleavage of the cell’s DNA in fragments that are multiples of 185 base pairs [42–44].

The morphological and histological findings of an antral follicle in the process of undergoing atresia include an increase in the number of pyknotic nuclei (apoptotic bodies) scattered in the membrana granulosa, granulosa cell detachment from the basal lamina which folds in on itself as the follicle shrinks. Penetration of the basal lamina (without degradation) by macrophages into the follicular antrum, cellular hypertrophy of the theca interna, the appearance of cellular debris in the antrum, disruption of the oocyte-cumulous cell connections, and eventually meiosis-like morphological changes of the oocyte followed by oocyte fragmentation [45, 46]. Note that pre-antral follicles utilize a different pathway to atresia and have a different histology [47, 48].

Antral stage follicular atresia begins with apoptosis in granulosa cells [49]. Healthy follicles may contain some apoptotic granulosa cells and apoptotic granulosa cells in large antral follicles do not impair or predict oocyte quality [44, 50, 51]. However, when the number or proportion of apoptotic granulosa cells exceeds a certain threshold, follicles become irreversibly atretic. Clinically, there are varying degrees of follicular atresia and in early stages, the process of becoming atretic can be suspended, most commonly by preventing further granulosa cell apoptosis by FSH rescue.

**Fig. 5:**
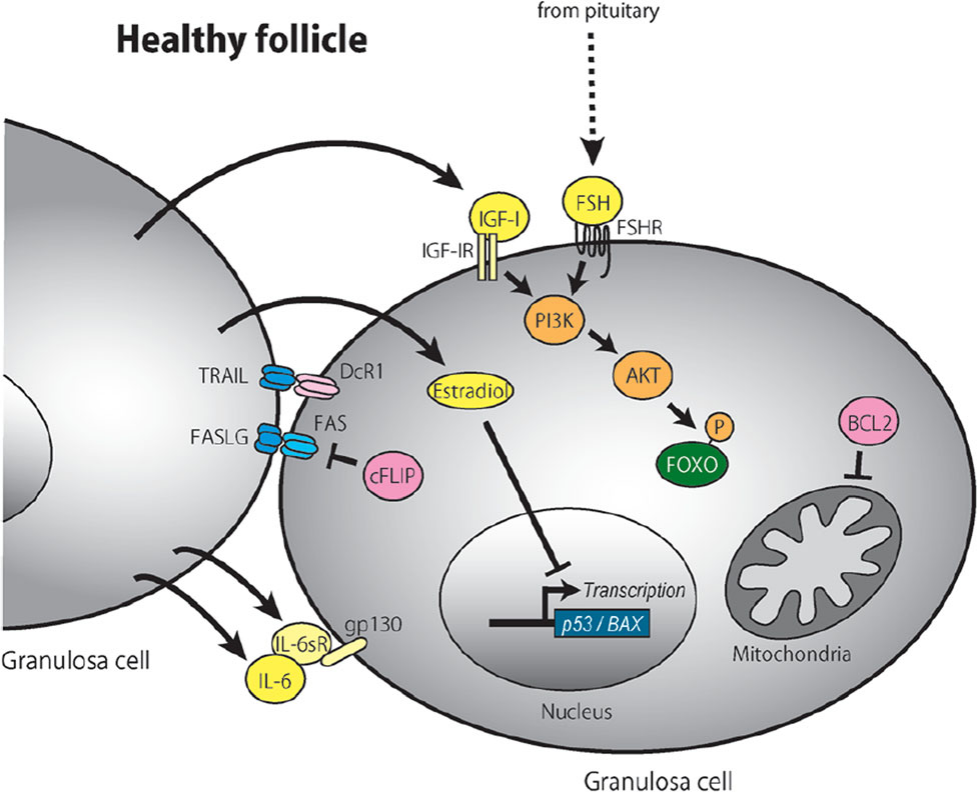
Schematic model of healthy growing granulosa cells. FSH secreted from the pituitary is essential for granulosa cell survival. IGF-1, estradiol, IL-6, and IL-6sR are expressed from granulosa cells, acting as survival factors of granulosa cells in a paracrine/autocrine manner. IGF-1 and FSH activate the intracellular PI3K/AKT pathway and phosphorylate FOXO transcription factors, which retain FOXO within the cytoplasm. Estradiol inhibits the transcription of proapoptotic genes *p53* and *Bax.* Death ligand receptors (FASLG-FAS and TRAIL-DcR1) are expressed on the cellular membranes. An intracellular molecule, cFLIP, inhibits the FASLG-FAS death signal. The TRAIL signal is inhibited by its decoy receptor, DcR1. BCL2 inhibits mitochondria-mediated apoptosis. From Matsuda et al. Follicular growth and atresia in mammalian ovaries: regulation by survival and death of granulosa cells. *J Reprod Dev* 2012; 58(1): 44–50

**Fig. 6:**
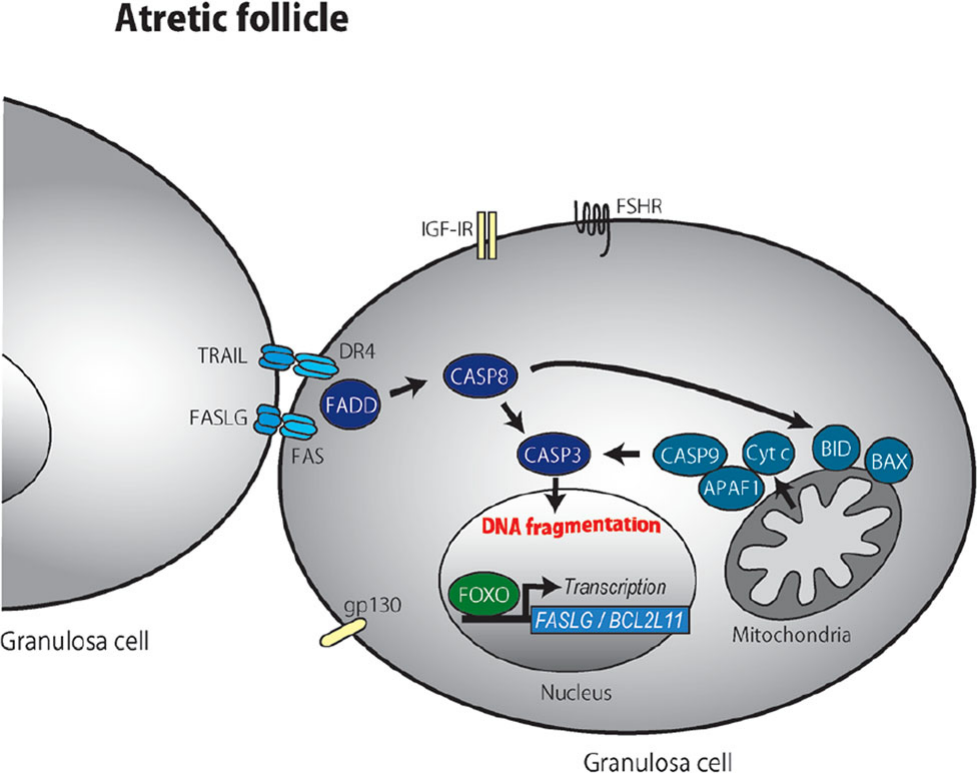
Schematic model of granulosa cells undergoing apoptosis. Cell survival signals such as FSH, IGF, estradiol, and IL-6 decline. As a result, FOXO is dephosphorylated and transferred into the nucleus, where it transactivates proapoptotic factors, FASLG and BCL2L11. Death ligand receptors (FASLG-FAS and TRAIL-DR4) expressed on the cellular membrane increase. Subsequent activation of intracellular signaling (FADD, CASP8, and CASP3) induces DNA fragmentation. CASP8 also stimulates the mitochondrial apoptotic pathway by inducing BID, BAX, Cyt c release, APAF1, and CASP9. From Matsuda et al. Follicular growth and atresia in mammalian ovaries: regulation by survival and death of granulosa cells. *J Reprod Dev 2012*; 58(1): 44–50

Granulosa cell apoptosis is a non-pathological process that is complexly regulated. There are two major pathways to granulosa cell apoptosis: a cell surface pathway and a mitochondrial pathway. Triggering either of these pathways involves the expression of one or more of a number of “death” inducing factors that are not suitably balanced by a number of “survival” or anti-apoptotic factors. Figures 5 and 6 illustrate these types of interactions, taking place in a granulosa cell, involved in maintaining a healthy follicle or in producing an atretic one [42, 43, 52]. Both experimentally and clinically exogenous FSH is a highly effective survival factor for granulosa cells and, in the late antral and pre-ovulatory follicles, enables follicles to avoid atresia.

To recapitulate, androgens are the primary anti-apoptotic factor in pre-antral and early antral follicles. Androgens indirectly are anti-apoptotic by increasing FSH receptors and through stimulation of mitosis. In addition, androgens directly inhibit apoptosis in granulosa cells by production of microRNA-125b which suppresses proapoptotic protein production [31]. FSH is the primary anti-apoptotic factor in larger follicles.

### Harmful effects of androgens on granulosa cells

High levels of androgens or exposure to androgens during late follicular development can promote follicular atresia [53–55]. Large atretic follicles have long been recognized to contain high androgen to estrogen ratios [33]. Female to male transsexuals demonstrate a 3.5-fold increase in atretic follicles in their ovaries [56]. Available data suggests that androgen levels are not increased in atretic follicles during spontaneous menstrual cycling; rather, estrogen levels are markedly decreased within an atretic follicle, resulting in a high androgen to estrogen ratio [33].

Chen et al. used a rat model to study the impact of dihydrotestosterone (DHT), an androgen that cannot be converted into estrogen, on large antral/pre-ovulatory follicles [57]. They attempted to model androgen levels consistent with those of women with polycystic ovarian syndrome (PCOS). In one short-duration experiment, they cultured granulosa cells from young rats. FSH treatment significantly enhanced cellular proliferation, but DHT suppressed this enhancement of proliferation with an accumulation of granulosa cells arrested in the G2/M phase of the cell cycle. DHT was found to inhibit FSH-induced phosphorylation of Akt. Phosphorylated Akt plays an essential role in granulosa cell survival and proliferation [40]. DHT treatment also promoted PTEN (phosphatase and tensin homolog deleted on chromosome 10) expression, which is involved in cell cycle arrest [58]. PPARɣ, one of the transcriptional factors upregulating PTEN expression [59], was also enhanced by DHT treatment. The increased mRNA expression of PTEN after DHT treatment was suppressed significantly by silencing RNA (siRNA) of PPARɣ. Since large follicles contain few or no androgen receptors, this impact of androgens must utilize a non-genomic (non-androgen receptor) pathway [57].

Chen et al. also looked at long-term in vivo effects of androgen treatment with DHT in large rat follicles [57]. Again, DHT caused a cellular proliferation in large follicles to be reduced, phosphorylated Akt to be suppressed, and PTEN and PPARɣ to be enhanced. Thus, they found the same impact of diminished granulosa cell proliferation by the same mechanism in both short-term granulosa cell culture with DHT and long-term DHT treatment of intact animals on large antral follicles.

However, granulosa cells from follicles from intact animals were also evaluated for apoptosis and no differences in the incidence of apoptosis by TUNEL (terminal deoxy-UTP nick end labeling) staining in follicles from DHT-treated and untreated animals were found. Nevertheless, these long-term DHT-treated animals had significantly fewer mature and ovulated follicles. The treated animals also had longer and fewer estrous cycles than controls. It is unclear exactly why apoptosis was not found in these large follicles with slowed development. TUNEL staining does not detect all apoptosis in granulosa cells [60]. However, since there were fewer ovulated follicles, this experiment suggests that follicles were lost from the ovulatory pool. Vendola et al. treated rhesus monkeys with DHT or testosterone and found that the number of small follicles increased threefold, but large antral follicles were similar to untreated controls [25]. Slow-growing follicles could remain viable and non-ovulatory by becoming cysts [61]. Follicles in long-term androgen-treated animal PCO models, which do not proceed to ovulate, often become cystic and eventually die using a pathway other than apoptosis [61]. Noorafshan et al. looked at the impact of letrozole treatment (21 days) in rats and found that granulosa cell numbers were decreased by 56% and there was a fivefold increase in the volume of ovarian cysts compared with controls [62]. Paixão et al. reviewed animal models from diverse species, using sundry androgens in short- and long-term androgen exposures and with androgen exposures at different times in the animal’s development [63]. Most studies of these models found an increase in both atretic and cystic follicles, but with some models having either atretic or cystic follicles dominant. In humans with severe PCOS, naturally occurring elevated androgens only rarely produce follicles larger than 10 mm with most follicles arresting below 7 mm and yet surviving for months [64].

In summary, high levels of androgens appear to directly slow follicle development in larger follicles by diminishing the ability of the granulosa cell mass in a follicle to proliferate enough to maintain an adequate FSH receptor population. As this process of mitosis slows, so does aromatase production, and resulting estradiol production. Slowing the growth of the membrana granulosa of a follicle decreases its access to apoptotic survival factors. If this decrease in mitosis is not enough to make the follicle atretic, it otherwise decreases the ability of that follicle to ever spontaneously ovulate. Cystic follicles may still contain non-apoptotic oocytes that could possibly remain useful in an IVF setting.

## Infertility treatments utilizing letrozole

Letrozole is not approved by the Federal Drug Administration (FDA) in the USA for any application in treating infertility or treating pre-menstrual women. Nonetheless, it is recommended as a first-line treatment for anovulation in women with PCOS by the American College of Obstetricians and Gynecologists [65, 66] and has been used innovatively in the applications discussed below. One of the goals of this paper has been to promote a better understanding of the physiological consequences of using letrozole in pre-menstrual women. The discussion of these infertility applications in this setting raises questions that best be answered experimentally, with appropriate care taken to protect patients as ethically and legally appropriate when undertaking medical research [67].

### Letrozole for fertility enhancement in the ovulatory patient

Letrozole is commonly used in ovulatory patients to increase their chance of becoming pregnant. Typical diagnoses include unexplained infertility, mild male factor, endometriosis, pelvic factor, and advanced maternal age. The objective of this therapy is to improve oocyte “quality,” to increase “control” of the ovulatory process, and/or to induce multiple follicle ovulation. The mechanism used is to increase a patient’s ovarian follicles’ exposure to FSH by decreasing the negative feedback impact of estradiol production by the ovary. Typically, treatment consists of 2.5 to 7.5 mg of letrozole taken by the patient on days 3 through 7 of their menstrual cycle [5–7]. Adjunctive therapies often used with letrozole include intra-uterine insemination (IUI) and ovarian hyperstimulation with gonadotropins.

Gougeon’s classes 5, 6, and 7 are defined by follicles 2 to 5 mm, 5 to 10 mm, and 10 to 16 mm in diameter, respectively (Fig. 4). Gougeon determined the amount of time it took for follicles to grow from one class into another [22]. In natural cycles, follicles grow at an average of 0.6 mm/day from 2 to 5 mm in diameter, 1 mm/day from 5 to 10 mm in diameter, and 1.2 mm/day from 10 to 16 mm in diameter. Pre-ovulatory follicles (> 14 mm) grow at a rate of 1.5 to 2 mm/day [68]. Therefore, a follicle that becomes 16 to 24 mm in diameter on day 12 of a menstrual cycle was 5 to 6 mm in diameter on cycle day 1, 6 to 8 mm in diameter on cycle day 3, 8 to 11 mm in diameter on day 5, and 10 to 15 mm in diameter on cycle day 7. Fauser and van Heuser [17] noted that women had healthy 2- to 5-mm-diameter antral follicles at the end of the luteal phase of their prior cycles.

As noted previously, follicles become FSH-dependent around 6 mm, aromatase expression begins around 9 mm, and aromatase expression rapidly accelerates when the follicle is 10 mm or more in diameter [17, 18, 24]. Thus, the initiation of letrozole on cycle day 3 commonly coincides with the availability of a 6- to 8-mm follicle which can become our desired ovulated follicle.

On the other hand, letrozole taken on day 3 increases intra-ovarian androgens in the “day 3 targeted” 6- to 8-mm follicle. The 6-mm follicle is equipped with a high level of androgen receptors and increased androgen levels at this time promote granulosa cell mitosis and induction of FSH receptors and help make the follicle more resistant to atresia. However, these androgen benefits dissipate as the follicle enlarges and androgen receptors sharply diminish. For example, in the 15-mm follicle on day 7, elevated androgen levels would decrease the rate of granulosa cell mitosis. Thus, the positive impact of androgens could be strengthened by starting letrozole earlier and, for some patients, the potential negative impact of increased androgens minimized by stopping it slightly earlier (in the patient likely to have a normally timed LH surge on cycle day 12).

Since early antral follicle exposure to FSH is likely not harmful, one could start letrozole on day 1 of a patient’s menstrual cycle to maximize the positive impact of androgens from letrozole on that patient’s cycle. The positive impact of androgens starts prior to that follicle’s antral life and thus, a clinician could also start letrozole during a prior cycle as long as the unintended impact on FSH was effectively managed (for example, by use of oral contraceptives to suppress FSH production). The above speculations are conjectural; to our knowledge, they have not been explored clinically.

### Letrozole and fertility preservation in the breast cancer patient

Breast cancer occurs in thousands of women under age 40 in the USA each year. Many of these women have yet to have completed their families. Some have yet to start. These women may seek help in preserving their fertility. The primary method used for fertility preservation involves some application of advanced reproductive technologies (ART), which requires treatment with exogenous FSH to develop as many follicles as safely possible. Oocytes harvested from these follicles are cryopreserved or fertilized and then cryopreserved. If the patient utilizes stored oocytes or embryos to achieve pregnancy in the future, the likelihood of successful pregnancy is correlated with the number of oocytes or embryos cryopreserved [69].

Since two-thirds of breast cancers are estrogen positive and estrogen promotes the growth of breast cancer cells [3, 4], a major concern with fertility preservation is the exposure of the patient to high doses of estrogen, as is typical during a controlled ovarian hyperstimulation in an ART setting. Oktay et al. showed that concomitant treatment with letrozole during the controlled ovarian hyperstimulation could be used to prevent high levels of estrogen production [8]. Typically, letrozole is started on day 2 or 3 of the menstrual cycle and exogenous FSH is administered starting 2 days later. Both are continued until oocyte maturity is triggered with hCG or GnRH agonist. A recent review by Rodgers et al. summarized twelve studies using this approach and found peak estradiol levels to average 337–829 pg/ml, which is only slightly higher than the peak estradiol levels found during natural cycles [9]. Since a letrozole dose of 2.5 mg decreases aromatase activity by greater than 99% in post-menopausal women [1, 15, 16], this finding about estradiol production highlights the differences in using letrozole in a pre-menopausal and a post-menopausal woman. In spite of high doses of letrozole, a pre-menopausal woman still produces a significant quantity of estradiol. Letrozole resides in the aromatase binding site until it is metabolized and then circulating letrozole, weakly bound to albumen, competes with free testosterone to occupy that site. The primary difference with respect to estrogen production between the post-menopausal women and the pre-menopausal women treated with FSH is the rapid multiplication of granulosa cells (beginning in the 6-mm follicle) and the massive increase in production of aromatase molecules in each of those granulosa cells (beginning in the 10-mm follicle). If the objective is to minimize estrogen production, one must maximize the ability of letrozole to compete for new aromatase binding sites. One way to help achieve this might be to administer the letrozole in a split dose rather than a single dose and to increase the letrozole dose as the cycle progresses (e.g., letrozole 2.5 mg two, three or four times a day depending on the women’s estradiol response).

Rodgers et al. also reviewed four studies that compared the number of oocytes retrieved when letrozole was added to conventional therapy and when it was not [9]. In two of the studies, the same number of oocytes was obtained. In the other two studies, the number of oocytes retrieved was significantly less (by 2 or 3 oocytes). This review pointed out that in one of those studies, less FSH was used in the letrozole group compared with the conventional group during the controlled ovarian hyperstimulation. As noted above, in the later part of an ovarian stimulation (when lead follicles are larger than 12–14 mm), androgen production in the ovary can have a negative impact on granulosa cell mitosis and follicle development. As follicle growth progresses, FSH may need to be increased to enhance granulosa cell mitosis and maintain healthy follicle development.

### Letrozole as adjunctive treatment in IVF for diminished ovarian reserve

A number of researchers have used androgens to try to improve the response to gonadotropins of patients with diminished ovarian reserve during IVF. One of the most noteworthy studies using letrozole was done by Garcia-Velasco et al. [10], who randomized 147 low IVF responders (less than four 16-mm follicles developed in a prior canceled IVF cycle) for a subsequent IVF cycle. The canceled cycle used high-dose gonadotropins after pituitary desensitization with GnRH agonist. The subsequent cycle used high-dose gonadotropins with GnRH antagonist started after the lead follicle reached 14 mm. The experimental group additionally received letrozole 2.5 mg daily for the first 5 days of gonadotropin stimulation. The cohort receiving letrozole had 6.1 oocytes retrieved compared with 4.3 oocytes in controls and had an implantation rate of 25% compared with 9.4% in controls. The pregnancy rate was not significantly higher, but the multiple birth rate was (46.7% vs 7.7%).

As in the first example, letrozole was started not only when the target antral follicles were 6 to 8 mm in diameter around the time that they were becoming FSH dependent but also when androgen receptors were starting to drop from their peak. Unlike the first example, excess FSH was available to compensate for any slowing down of granulosa cell mitosis as the follicles enlarged in the face of elevated androgens. However, the number of target size antral follicles likely could have benefited and their cellularity enhanced by elevating androgens earlier in these follicles by starting letrozole earlier in the cycle. Academically, it is hard to hypothesize a downside in starting letrozole on day 1 of this type of cycle instead of on cycle day 3. Androgens also enhance pre-antral follicle growth [24, 27] and enable pre-antral and very early antral follicles to avoid atresia. The time required for pre-antral follicles to develop into 2-mm antral follicles is about 3 months. The antral cavity (0.1 to 0.2 mm) forms about 65 days prior to the formation of the 2-mm follicle [22]. In theory, some patients with severely diminished ovarian reserve might benefit from a treatment that increases intra-follicular androgens as early as the pre-antral follicle development stage.

The use of letrozole to augment IVF response in women with presumed normal ovarian reserve has also been studied. Haas et al. randomized 174 women to routine antagonist ovarian hyperstimulation with and without 5 mg of letrozole from the start of gonadotropins to the ovulation trigger day [70]. Patients at high risk for “clinical” ovarian hyperstimulation syndrome (OHSS) had ovulation triggered by GnRH agonist, but the choice of fresh or frozen embryo transfer was up to the physician. Assessing the letrozole group compared with traditional therapy, the number of retrieved oocytes (14.5 versus 10) and the number of blastocysts (4.0 vs 2.7) were higher. There was no difference in the cumulative pregnancy rate at the time of publication, but the influence of embryo cryopreservation made this difficult to evaluate. Since vascular endothelial growth factor (VEGF), strongly implicated as a possible cause of OHSS, is increased with the use of letrozole in humans [71], the potential for OHSS in patients with a high ovarian reserve may be a concern for women with normal or high ovarian reserve.

Garcia-Velasco et al. [8] and Hass et al. [71] both showed that letrozole taken during the first 5 days of gonadotropins or until ovulation triggering during IVF increased androgen levels in follicular fluid of pre-ovulatory follicles. However, at this time in follicular development, virtually no androgen receptors are present in granulosa cells [18, 19].

Several research studies have used exogenous testosterone to improve ovarian function in women prior to IVF [72]. This is likely to be less effective in functionally increasing intra-ovarian androgen levels in the small antral follicle than the use of letrozole. Kristenson et al. measured testosterone levels in the follicular fluid of small antral follicles taken from 286 women with a mean age of 28 who were undergoing ovarian tissue preservation [18]. They found that their testosterone levels ranged from about 200 to 300 nmol/ml (approximately 5000 to 8000 ng/ml), which is significantly higher than serum testosterone levels in either normal or PCO women [18]. A related critique could be directed against the idea that early use of letrozole may increase intra-follicular androgens as these must be derived from substrate that the follicle is trying to convert to estrogens in a setting of more limited aromatase. Exogenous testosterone may be most effective at a time more distant from the development of the small antral follicle (presumably when intra-follicle androgen and estrogen levels are low), whereas letrozole may be more effective temporally closer to a time of more rapid follicle development.

### Letrozole priming for in vitro maturation

IVF utilizing in vitro maturation (IVM) involves harvesting oocytes from small to medium antral follicles and maturing them in vitro before fertilization. Oocytes are harvested from follicles less than 13 mm in diameter [73]. Although IVM can be done without the use of any supplementary medications, many programs utilize low doses of FSH or “priming” to enhance the likelihood of live birth after an IVM treatment cycle. The use of hCG given 38 h prior to retrieval is also referred to as priming. Likely, the most common use of priming involves treatment with 150 U FSH for 3 days given starting on cycle day 3 [73]. The objective of priming is to make the oocytes more competent to become embryos and eventually live births. Follicles at this time have FSH receptors and can benefit developmentally from available FSH. Most follicles at the start of an IVM cycle will be in the FSH responsive stage, rather than the FSH-dependent stage. Only the larger of these antral follicles will be synthesizing aromatase. That an increased number of granulosa cells in a follicle could enhance oocyte competence is a reasonable hypothesis.

Rose et al. have used letrozole (at times with a low dose of FSH) as a method of “priming” with IVM with the objective of trying to enhance granulosa cell mitosis to both make follicles larger prior to oocyte harvesting and their oocytes more competent [12]. In particular, letrozole was given starting menstrual cycle day 3 for 5 days, followed by FSH at a dose of 25 to 75 U starting on cycle day 7.

All applications of FSH and letrozole for priming have served to increase the granulosa cell mass of a follicle and have primarily used FSH action as the mechanism to do this. This may represent a lost opportunity, perhaps depending on the patient population. Notionally, letrozole can increase the antral follicle count, granulosa cellularity, granulosa cell FSH responsiveness, and follicle resistance to atresia by increasing androgen availability in the early antral follicle. This benefit potentially seems especially valuable in applying IVM to non-PCOS patients. Exactly how letrozole affects small antral follicles in PCOS patients has not been explored.

The effectiveness of letrozole in increasing endogenous FSH by interrupting the negative estrogen feedback loop is well-established. All antral follicles have granulosa cells with FSH receptors, even when these cells are primarily androgen dependent. Thus, letrozole could positively impact follicles in an IVM cycle by FSH-mediated mechanisms until oocyte retrieval. Small antral follicles may also be positively impacted by an increased androgen effect. Letrozole could be used to enhance granulosa cell number and FSH responsiveness at the start of an IVM cycle. As the cycle progresses, follicle growth will also depend on the balance between intra-follicle androgens and circulating FSH. Supplemental FSH could also be used to tip this balance toward desired follicle size by mitigating adverse androgen effects in the last few days before retrieval.

## Conclusions

Letrozole is a targeted aromatase inhibitor that has primarily been used in post-menopausal women. In that setting, its actions are well understood. Recently, it has been applied to the infertile pre-menopausal women because of its ability to interrupt the negative feedback action of estrogen produced by the ovary on FSH production. However, the developing ovarian follicle is undergoing rapid endocrinological changes in the last 2 weeks of development prior to its ovulation and only a portion of these changes are a consequence of its sensitivity to FSH. The health of the small antral follicle is driven primarily by androgens, which contribute to granulosa cell mitosis, sensitivity to FSH, and resistance to atresia, whereas the health of the late antral follicle is driven primarily by FSH. A confounding issue is that elevated androgens in the late antral to pre-ovulatory follicle have a negative impact on follicle health and lead to atresia and cystic follicle formation.

Basic science information suggested that current applications of letrozole to infertility treatments may be wasting some of the primary benefits available in using letrozole to promote follicle development. Four applications of letrozole to infertility that have appeared in the medical literature were reviewed. These applications of letrozole were examined and various questions were raised about how letrozole could be more effectively used to help patients in these settings. The common theme underlying this discussion was that increased developmental benefits can be obtained by starting letrozole as early as possible and potential adverse androgen effects in the late cycle can be mitigated by ensuring adequate FSH availability in the latter part of follicle development. Many open questions remain, but basic science research supports the optimism that consideration of androgen impacts on follicle development could lead to more efficient infertility therapies.
